# The Src Homology 2 Domain-Containing Adapter Protein B (SHB) Regulates Mouse Oocyte Maturation

**DOI:** 10.1371/journal.pone.0011155

**Published:** 2010-06-16

**Authors:** Gabriela Calounova, Gabriel Livera, Xiao-Qun Zhang, Kui Liu, Roger G. Gosden, Michael Welsh

**Affiliations:** 1 Department of Medical Cell Biology, Uppsala University, Uppsala, Sweden; 2 Laboratory of Development of the Gonads, UMR-U967, INSERM/CEA/Paris Diderot-Paris 7 University, Fontenay aux Roses, France; 3 Department of Medical Biochemistry and Microbiology, Uppsala University, Uppsala, Sweden; 4 Department of Medical Biochemistry and Biophysics, Umeå University, Umeå, Sweden; 5 Center for Reproductive Medicine and Infertility, Weill Medical College, Cornell University, New York, New York, United States of America; Cincinnati Children's Research Foundation, United States of America

## Abstract

SHB (Src homology 2 domain-containing adapter protein B) is involved in receptor tyrosine kinase signaling. Mice deficient in the *Shb* gene have been found to exhibit a transmission ratio distortion with respect to inheritance of the *Shb* null allele among offspring and this phenomenon was linked to female gamete production. Consequently, we postulated that *Shb* plays a role for oocyte biology and thus decided to investigate oocyte formation, meiotic maturation, and early embryo development in relation to absence of the *Shb* gene. Oogenesis was apparently accelerated judging from the stages of oocyte development on fetal day 18.5 and one week postnatally in *Shb* −/− mice; but in adulthood ovarian follicle maturation was impaired in these mice. Completion of meiosis I (first polar body extrusion) was less synchronized, with a fraction of oocytes showing premature polar body extrusion in the absence of *Shb*. *In vitro* fertilization of mature oocytes isolated from *Shb* +/+, +/− and −/− mice revealed impaired early embryo development in the −/− embryos. Moreover, the absence of *Shb* enhanced ERK (extracellular-signal regulated kinase) and RSK (ribosomal S6 kinase) signaling in oocytes and these effects were paralleled by an increased ribosomal protein S6 phosphorylation and activation. It is concluded that SHB regulates normal oocyte and follicle development and that perturbation of SHB signaling causes defective meiosis I and early embryo development.

## Introduction

Understanding the processes regulating oocyte maturation is a topic of potential importance for assisted reproductive technology for treating infertility as well as in understanding molecular foundations of oocyte biology and pathology. The influence of external hormonal factors as well as the intricate interplay between oocytes and the surrounding somatic follicle cells have been extensively investigated, though many details of the regulation of these processes remain elusive. The challenge for discovering control mechanisms lies in the difficulty of investigating the cumulus-oocyte complex which is a developmental unit, implying that oocytes in isolation do not faithfully reflect biology in situ.

Targeted gene deletion provides an excellent strategy for studying follicle biology and creating models of human reproductive disorders [1], [2]. Knocking out genes coding for hormones and paracrine factors has been particularly successful. For example mutations of genes encoding growth-differentiation factor 9 and bone morphogenic protein 15 [3], [4], [5], [6] have revealed that they serve as oocyte secreted paracrine factors operating in concert with steroids and follicle-stimulating hormone (FSH) to stimulate granulosa cell growth and differentiation. Another example is the phosphoinositide-3′-phosphate phosphatase, PTEN (phosphatase and tensin homolog deleted on chromosome ten), which regulates follicular activation and in whose absence oocyte growth is prematurely activated leading to premature ovarian failure [7].

SHB (Src homology 2 domain-containing adapter protein B) is a ubiquitously expressed SH2-domain adapter protein that has not previously been investigated for a role in oocyte maturation. It is involved in receptor tyrosine kinase signaling in many tissues by generating signaling complexes in response to receptor activation [8], [9]. Furthermore, SHB has been found to interfere with the actions of the ubiquitin ligase atrophin-interacting protein 4 (AIP4) [10]. SHB has pleiotropic effects and the responses that it transduces depend on both the stimulatory factor and the cell type in which SHB signaling is active. It participates in the regulation of apoptosis, cytoskeletal physiology, proliferation and differentiation [8], [11], [12], [13]. Embryonic stem cells deficient in *Shb* showed altered patterns of differentiation in both mesodermal and endodermal lineages [11], [12], [14], [15].

A *Shb* gene knockout mouse was recently generated both on a mixed genetic background (FVB/J-C57Bl/6-129Sv) and in C57Bl/6 mice for studying its role in vivo [16]. Although heterozygotes were viable, *Shb* −/− embryos and mice could not be created on a C57Bl/6 background but only on the mixed genetic background, and consequently, the *Shb* knockout is fertile only in the latter case. Interestingly, there was a transmission ratio distortion because the *Shb* null allele was preferentially transmitted from *Shb* +/− mothers. Additionally, there was relative loss of *Shb* −/− embryos that could have arisen at implantation or from embryo resorption and severe developmental anomalies. Postnatally, aberrations were observed in *Shb* knockout mice, including defective angiogenesis [17], impaired glucose homeostasis [18] and diminished beta cell stress responses [19]. We hypothesized that the transmission ratio distortion of the *Shb* null allele reflected abnormalities in oocyte maturation, for which we found evidence in the present study of *Shb* deficient mice.

## Results

### Oocyte/follicle morphology in late fetal, post-natal and adult ovaries

The previously observed transmission ratio distortion and the embryonic malformations in offspring from *Shb* +/− or −/− mice raise the possibilities that oocyte development and/or function are altered in this mutant. Oocytes enter meiosis I during late fetal development (around 18.5 dpc in the mouse) and form primordial follicles together with somatic cells around birth. A few of these develop to become primary follicles at one week after birth. Follicle maturation further progresses by development to antral follicles when the female mouse reaches a reproductive age, of which a few ovulate in each estrous cycle. Consequently, we decided to study oocyte/follicle morphology at these stages of ovarian development in relation to the *Shb* gene mutant classifying germ cells into primary oocytes, primordial follicles, primary follicles and antral follicles according to the shape and number of layers of somatic cells surrounding the oocytes.

**Figure 1:**
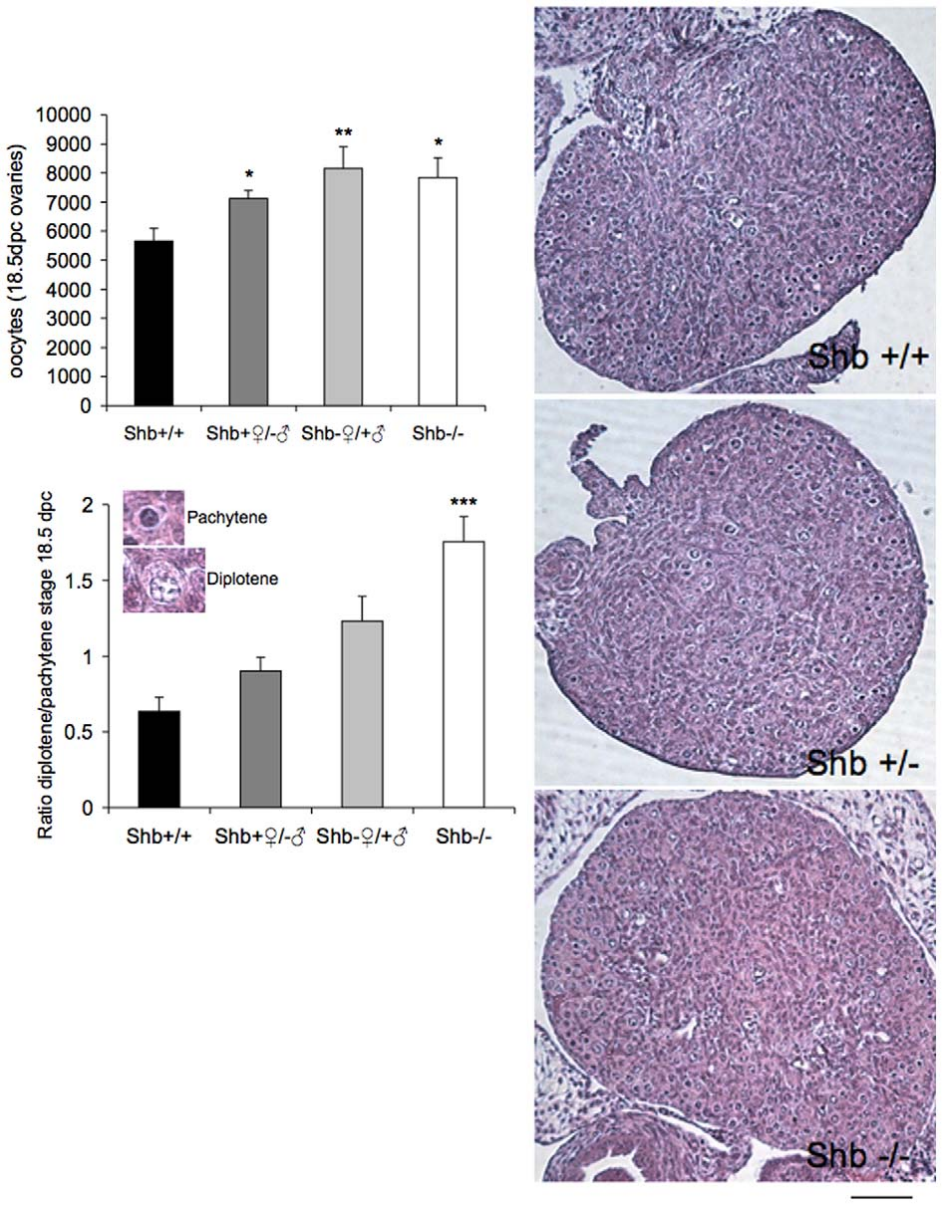
Number of primary oocytes and relative degree of maturation in fetal ovaries (18.5 dpc). Ovaries were subjected to morphological analysis determining oocytes in the pachytene and diplotene stages. Representative images of oocytes and ovaries are included in the figure (scale bar indicates 100 µm). Values are for four genotypes including *Shb* +/− (wild-type mother) and *Shb* −/+ (wild-type father). Means ± SEM for n = 5 (+/− and −/+) and n = 10 (+/+ and −/−) ovaries. *, ** and *** indicate p<0.05, 0.01 and 0.001, respectively, compared with wild-type control by ANOVA (Fisher LSD test).

**Figure 2 pone-0011155-g002:**
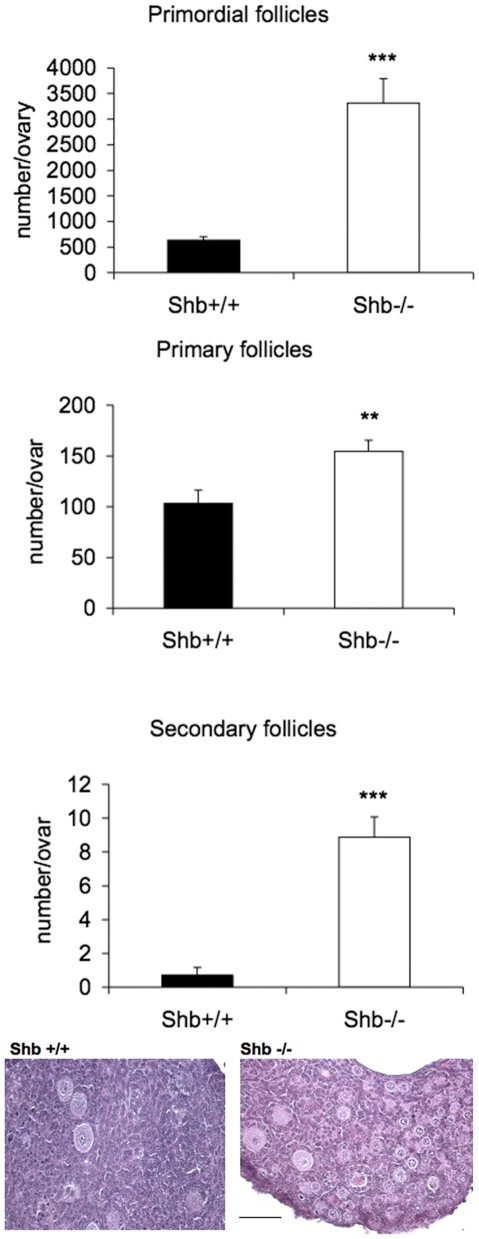
Follicle development of 18.5 dpc fetal ovaries from *Shb* +/+ and *Shb* −/− mice after culture for 7 days. Fetal ovaries were cultured for 7 days and the organ explants morphology examined. Representative images are included in the figure (scale bar indicates 100 µm). Means ± SEM are given for n = 6–8. ** and *** indicate p<0.01 and <0.001, respectively compared with *Shb* +/+ using a Student's t-test or a Mann-Whitney rank sum test.

There was a significant increase compared to wild-type controls in the number of oocytes in *Shb* −/− fetuses at 18.5 dpc (Fig. 1). Most wild-type oocytes were, as expected at this age, at the pachytene stage of meiosis I. The lower panel of Fig. 1 shows an increased ratio of diplotene to pachytene oocytes in *Shb* −/− ovaries, with heterozygotes intermediate between them and controls. These findings led to the study of organotypic cultures of ovaries at the same ages to isolate oogenesis from systemic effects that may differ according to the genotype. The conditions of culture did not fully reproduce the physiological in vivo maturation process because fewer germ cells survived, yet the degree of organ differentiation was similar after a week. There was a significant increase in number of follicles at all stages in *Shb*−/− ovaries compared to wild-type which amounted to several fold at primordial and even secondary stages (Fig. 2).

**Figure 3:**
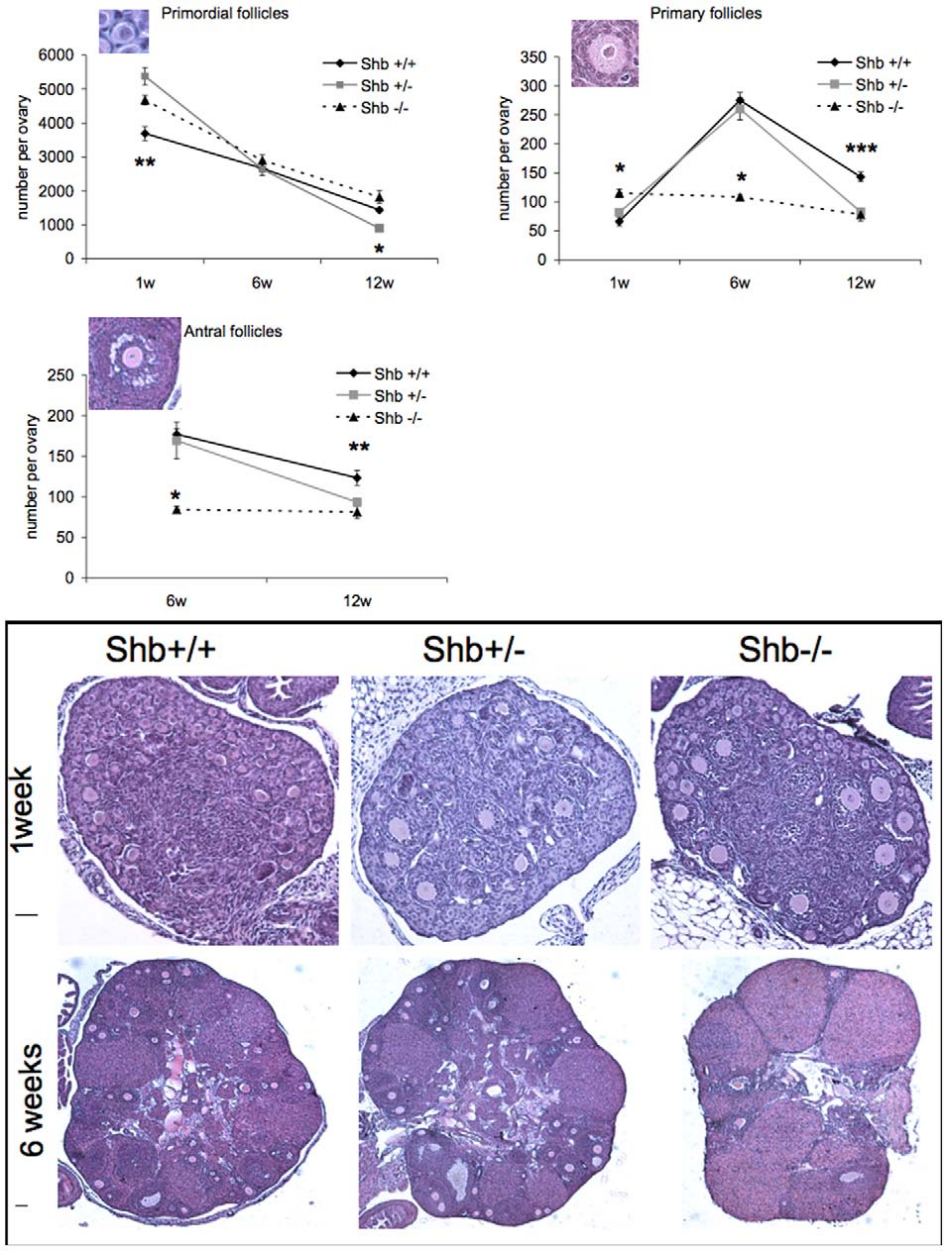
Number of primordial, primary and antral follicles from Shb+/+, Shb+/− and Shb−/− female mice at 1, 6 and 12 weeks of age. Morphological analysis of ovaries at the different ages. Representative images are included (scale bars to the left indicate 50 µm). Values are means ± SEM (n = 10 except +/+ at week 1 where n = 7). * denotes a p<0.05; ** p<0.01; *** p<0.001 comparing *Shb* −/− with +/+ using ANOVA (Fisher LSD test at one and 12 weeks and Kruskal-Wallis variance on ranks at six weeks) or +/− primordial follicles at 12 weeks with +/+ (Kruskal-Wallis variance on ranks).

In line with the increased number of oocytes in 18.5 dpc fetuses, the number of primordial follicles was higher in *Shb* +/− and −/− ovaries at one week of age compared to wild-type mice (Fig. 3), although the subsequent rate of disappearance appeared to be increased in the *Shb* +/− ovaries as suggested by the fact that at 12 weeks they had the least number of follicles. Primary follicles, the first stage of follicle growth, were more abundant in *Shb* −/− ovaries at week 1 (Fig. 1), but this was the only stage at which growing follicles (primary and antral) were elevated compared to the other two genotypes. The difference was revealed more strikingly when the data were expressed as ratios of primary or antral to primordial stages at weeks 6 and 12 when they were significantly lower in the knockout (Supporting Fig. S1). The ovarian weights at weeks 1 and 6 corresponded to the relative number of growing follicles estimated in tissue sections without exhibiting any direct relationship to body weight, although *Shb* −/− mice were heavier than the other genotypes at week 1 (Supporting Table S1).

Taken together, the data in Figs. 1–3 suggest that absence of *Shb* promotes early (pre- and post-natal) oocyte development whereas that at reproductive ages is retarded.

### Mature oocyte analyses

**Figure 4:**
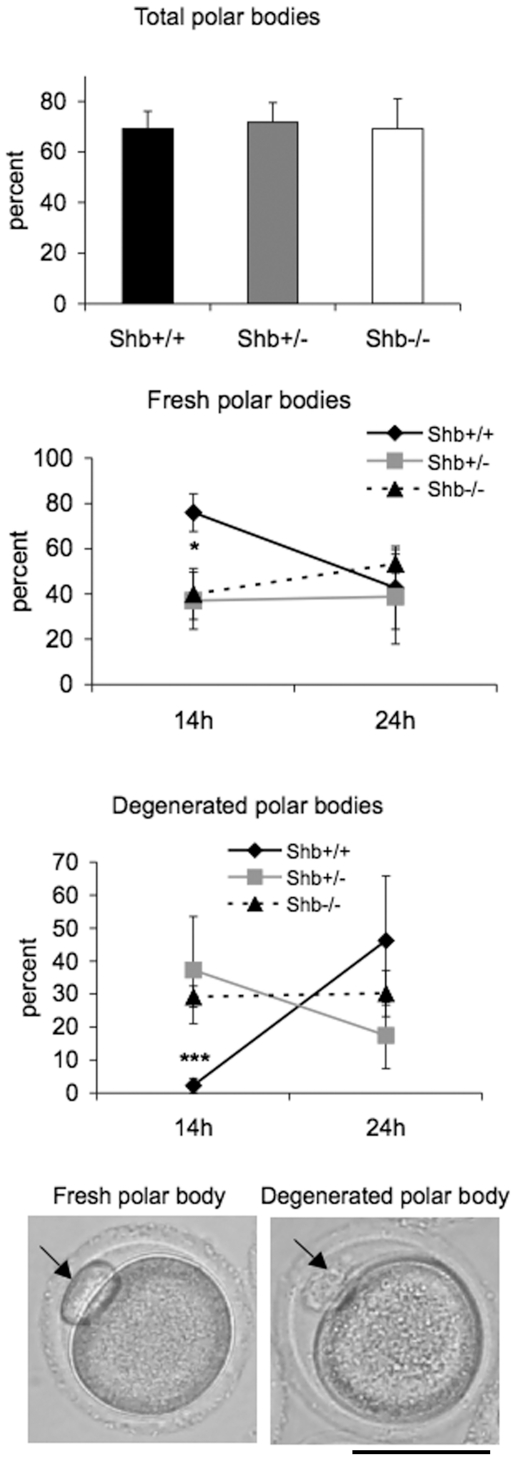
Extrusion of polar body after superovulation. Percentage oocytes with total polar bodies (fresh and degenerated) at 14 h after hCG injection, as well as fresh and degenerated polar bodies at 14 and 24 h after hCG injection are given as means ± SEM for four to five mice. * and *** indicate p<0.05 and 0.001, for *Shb* +/+ and *Shb* −/− oocytes, respectively, using a Student's t-test. Scale bar indicates 50 µm.

Mammalian oocytes remain at prophase of meiosis I from late embryonic stages until ovulation, when meiosis resumes. Resumption of meiosis and progression to metaphase I is characterized by germinal vesicle breakdown (GVBD), chromosome condensation and segregation, and is then followed by extrusion of the first polar body. It seemed appropriate to study the effect of the *Shb* gene on these events as well since the *Shb*-dependent effects on reproduction could result from events occurring at this stage of oocyte development. As an indicator of whether SHB has an effect on the timing of oocyte maturation around ovulation, first polar body extrusion and its degeneration were assessed at 14 and 24 h after superovulation. While the percentages of oocytes containing polar bodies that could be detected (total fresh plus degenerated) at 14 h were similar in the three groups of *Shb* oocytes (Fig. 4), *Shb* +/− and *Shb* −/− oocytes had significantly more degenerated polar bodies compared to wild-type controls, although the difference disappeared at 24 h. These results suggest less synchronized completion of meiosis I with a fraction of oocytes displaying accelerated resumption of meiosis as a consequence of *Shb* inactivation.

**Figure 5:**
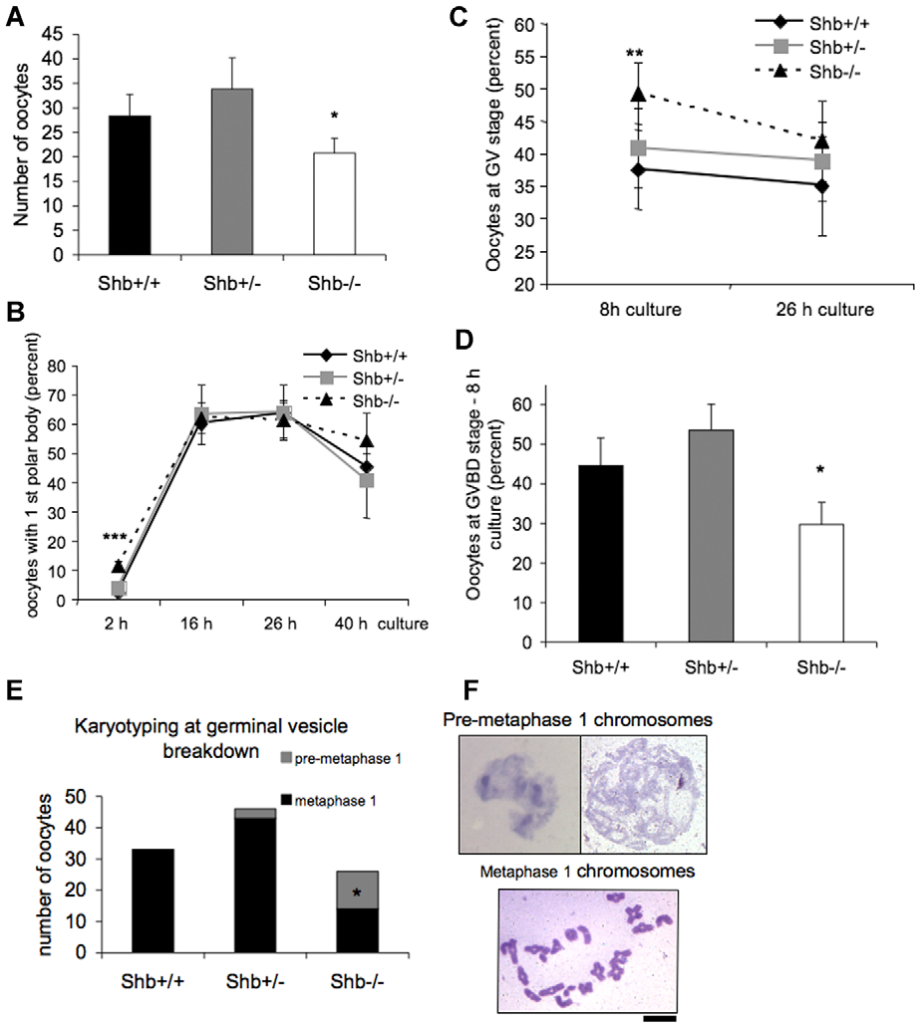
Oocyte maturation and chromosome karyotyping. Oocytes were mechanically recovered from ovaries 42 h after PMSG injection followed by culture for 2, 8, 16, 26 and 40 h. In A, the number of retrieved oocytes is given. B shows polar body extrusion. C depicts the percentage of oocytes in the GV (germinal vesicle) stage and D the corresponding GVBD (germinal vesicle breakdown) values. E shows the quantification of chromosomal characteristics after karyotyping at the GVBD stage and F provides representative images of such chromosomal preparations. Means ± SEM per mouse for n = 15 in A are given. In B, n = 11 at 2 h, n = 6 at 26 hours and n = 3 at 16 and 40 h. * and *** indicate p<0.05 and p<0.001, respectively, when compared with wild-type control using a Wilcoxon signed rank test. In C and D, means ± SEM for n = 11 at 8 hours and n = 5 at 26 hours. * and ** indicate p<0.05 and 0.01, respectively compared with wild-type control using a paired t-test. In E, the number of oocytes karyotyped from 5 separate experiments is given, including those in metaphase MI and pre-metaphase MI. * indicates p<0.05 when compared with wild-type control using a Chi-square test. Scale bar indicates 10 µm.

To obtain a time-course of oocyte maturation following PMSG injection, oocytes were mechanically released and cultured for different time periods. The number of GV oocytes that were recovered with this procedure at 42 h after PMSG injection from 2 to 4 month-old ovaries was significantly lower in *Shb* −/− mice than controls (Fig. 5A). This result was comparable to counts of antral follicles in the ovaries of corresponding genotypes at the same age (Fig. 3). When the oocytes were cultured for 2 hours (corresponding to 44 h after PMSG injection), *Shb* −/− oocytes had an increased prevalence of polar body extrusion (Fig. 5B) compared to controls, this observation being in line with what was observed at 14 h in the superovulation study (Fig. 4). The difference in polar body extrusion disappeared at later time points and this is also in agreement with the findings in Fig. 4 at 24 h after hCG injection.

To directly determine whether SHB had any effect on resumption of meiosis in oocytes, we examined the rates of GV (germinal vesicle) and GV breakdown (GVBD) in oocytes isolated from ovaries after PMSG treatment. Results are shown in Fig. 5C-D. At 8 h of culture (50 h after PMSG injection), we detected significantly more oocytes at the GV stage and fewer oocytes with GVBD in *Shb* knockouts than in controls. Karyotyping oocytes at the GVBD-stage revealed condensation of chromosomes at meiotic metaphase I. Fig. 5E-F display the results of karyotyping, in which in half of the chromosomes were in pre-metaphase I in *Shb* knockout oocytes. The combined results in Figs. 4–5 indicate reduced meiotic synchronization of *Shb* knockout oocytes with a certain fraction of oocytes showing accelerated meiosis I and others exhibiting a delayed response.

### *In vitro* fertilization (IVF) and blastocyst morphology

**Figure 6:**
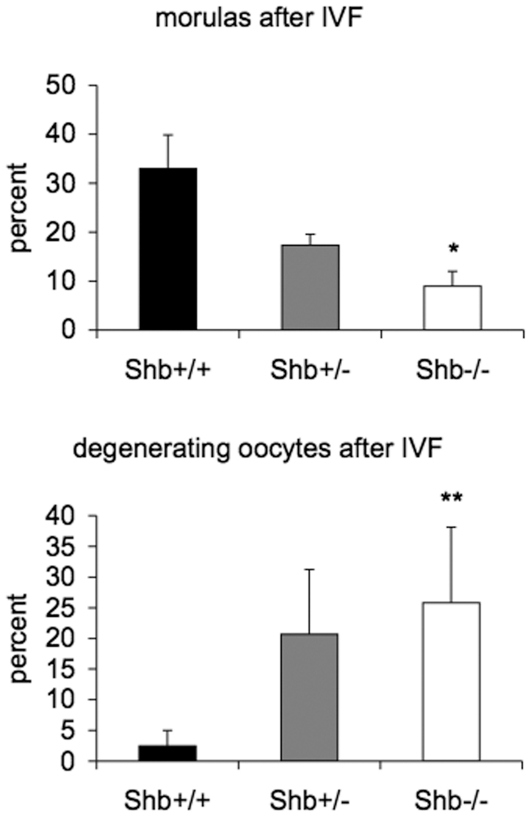
Embryo development after in vitro fertilization. Fertilized oocytes developing to the morula stage and those undergoing degeneration were scored as percentages in each group of collected oocytes. Means ± SEM in four separate experiments are given. * and ** indicate p<0.05 and 0.01, respectively when compared with wild-type control using a paired Student's t-test.

The effect of *Shb* deficiency on early embryo development was studied after in vitro fertilization and in blastocysts retrieved from pregnant mice at 3.5 dpc since an altered oocyte maturation before and after ovulation could have an effect on these processes. Oocytes were scored for degeneration or successive stages of development to the morula (Fig. 6) after a culture time of 24 h that followed the 4 h IVF period. The percentage zygotes that developed to the morula stage was lower than expected and this may be due to the mixed genetic background currently used compared with the F1(C56Bl/6xA2G) background normally adopted for IVF studies. Significantly fewer oocytes reached the morula developmental stage from fertilized *Shb* −/− oocytes compared to wild-type controls. In addition, significantly more *Shb* knockout oocytes degenerated after the 24 h culture period. The numbers of oocytes that showed no signs of degeneration or mitosis were similar in the +/+, +/− and −/− groups (42–46%). Table 1 shows the genotypes of oocytes/early embryos obtained from *Shb* +/− oocytes in vitro fertilized with wild-type sperm. The transmission ratio distortion was again observed with a relative increase in the number of oocytes carrying the mutated allele, but the wild-type allele was preferentially present in the embryos reaching the morula stage compared with those retarded at the 2–8 cell stage.

**Table 1:** The genotype ratio was distorted after IVF with oocytes from *Shb* +/− mothers and *Shb* +/+ sperm.

Stage	*Shb* +/+	*Shb* +/−
2–8 cell stage	1* (7%)	13 (93%)
Morula	9 (36%)	16 (64%)
Unfertilized	15 (31%)	34 (69%)
Degenerated	7 (21%)	27 (79%)

Oocytes from +/− mothers were isolated, in vitro fertilized with +/+ sperm and differentiated in vitro as in Fig. 6. At 24 h after IVF the early embryos/oocytes were classified and genotyped. The absolute and relative (in percent) +/+ vs +/− embryos/oocytes are given for each category. * indicates p<0.05 when compared with the corresponding number of morulas using the Chi-square test. The total percentage +/+ genotype was 26% for 122 observations.

**Figure 7:**
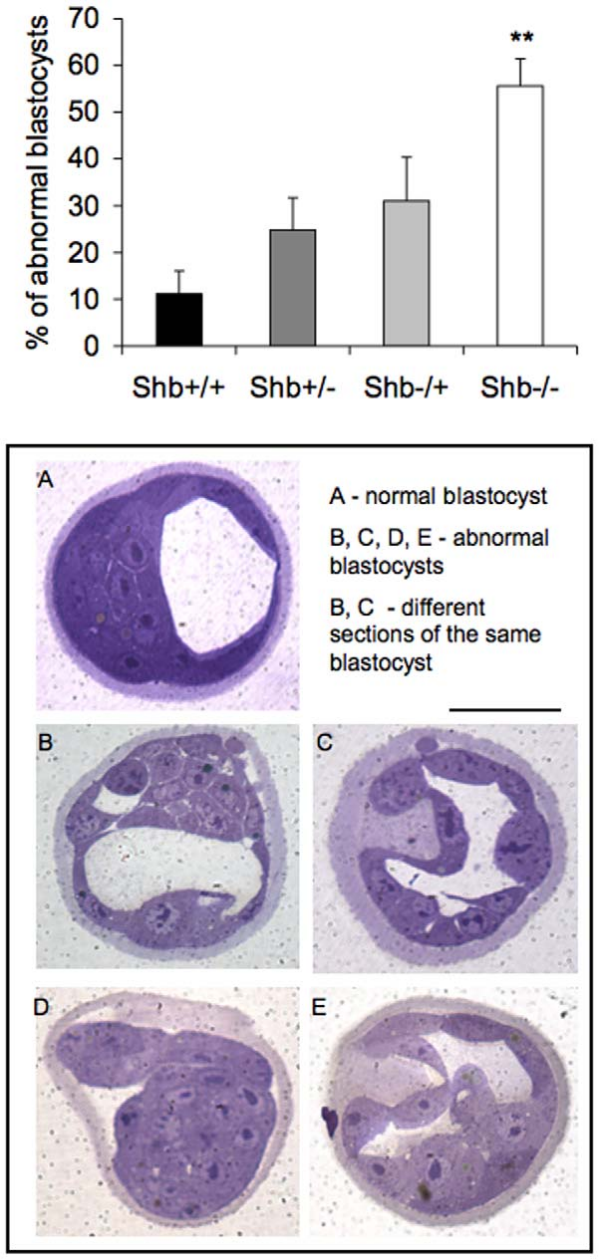
Blastocyst morphology at 3.5 dpc. Percentage abnormal embryos was determined in each litter and expressed as means ± SEM for five experiments. +/− is wild-type mother and −/+ is wild-type father. ** indicates p<0.01 when compared with wild-type control using Student's t-test with Bonferroni correction. A shows wild-type blastocyst, B-E *Shb* −/− blastocysts. Scale bar indicates 100 µm.

The characterization of early embryo development in vitro was extended further by analysis of blastocyst morphology at 3.5 dpc (Fig. 7). An increased proportion of blastocysts from the *Shb* knockout were less mature (the blastocoele was smaller or not present), they showed abnormalities in the inner cell mass with intercellular spaces between cells and abnormalities in the trophoectoderm with thickened and bulging cells. The genotype of the embryo was primarily responsible for these abnormalities, and no difference in the characteristics of oocytes from *Shb* +/+ or −/− mothers was observed, suggesting that Shb is required for early embryonic development as well.

Taken together, the data in Figs. 6–7 suggest impaired early embryonic development as a consequence of *Shb* deficiency.

### *Shb* and oocyte signaling

**Figure 8:**
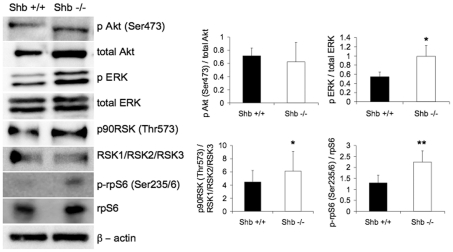
Akt, ERK, RSK and rpS6 activities/phosphorylation. Oocytes were isolated from neonatal *Shb* +/+ and −/− mice and subjected to Western blot analysis. Phosphorylation was related to total protein content on the same blot and determined by densitometric analysis. Values are arbitrary units expressed as means ± SEM. n = 3 for RSK, n = 4 for Akt, n = 5 for ERK and n = 6 for rpS6. * and ** indicate p<0.05 and 0.01, respectively using a paired t-test.

SHB is a multifunctional adaptor protein potentially affecting numerous signaling pathways which were investigated in postnatal ovaries to obtain a mechanistic explanation for the alterations in follicle and oocyte development observed. Expression of p-ribosomal protein (rp) S6 (Ser235/236), total rpS6, p-Akt (Ser473), total Akt, p-ERK (Thr202/Tyr204), total ERK, p90RSK (Thr573) and total RSK1/RSK2/RSK3 was determined by Western blot analysis. There was no effect on Akt phosphorylation (Fig. 8), but ERK was activated in the absence of SHB. Since RSK (p90) is a kinase downstream of ERK this protein could be activated in response to ERK activation, as indeed it appeared from phosphorylation at an activating site (Fig. 8). RpS6 is one of the targets of the RSK kinase and since rpS6 phosphorylation previously was found to correlate with premature oocyte maturation phosphorylation of this protein was also studied. Interestingly, rpS6 phosphorylation was augmented in the absence of SHB, suggesting stimulation of the ERK-RSK-rpS6 signaling pathway.

The SHB-dependent alterations in oocyte signaling are likely to contribute to the reproductive aberrations presently observed.

## Discussion

Evidence presented above shows that absence of SHB has significant effects on follicle development and/or oocyte maturation. Whereas SHB depletion promotes early oocyte development, its absence retards oocyte maturation in the adult. This divergent action is consistent with the pleiotropic nature of SHB as an adapter protein participating in numerous responses [8]. In addition, most of the present findings do not distinguish between systemic, ovarian or cell autonomous effects and this might contribute to the diversity of the responses. *Shb*-null pups have an increased weight which might have an impact on the developmental stage of the ovaries. However, it seems unlikely that this is the sole explanation for the observed accelerated oocyte differentiation for several reasons. Firstly, the effects were reproduced in organ culture, reducing the influence of systemic factors. Secondly, the mother's genotype (+/+ or −/−) played no role for the outcome of maturation in the heterozygote fetuses. Finally, the number of oocytes was increased in the knockout both before and after birth, thus suggesting that the process of generating primary oocytes is intrinsically enhanced by the absence of SHB and thus not related to maturity as such.

In adult ovaries, the absence of *Shb* exerts detrimental effects on follicle maturation with a relative reduction in the number of antral follicles and this could be the consequence of an action of SHB in germ and/or somatic cells. Follicle maturation is a consequence of an intricate interplay between granulosa/cumulus cells and the oocyte where granulosa/cumulus cells stimulate oocyte growth by producing the c-Kit ligand [20], [21], and the oocyte reciprocates by releasing growth differentiation factor 9 and bone morphogenic protein 15 [6], [22], [23]. In addition, c-Kit signaling has been shown to play a role in primordial germ cell migration, proliferation and survival [24], [25]. It is currently unknown whether signaling from the c-Kit tyrosine kinase receptor involves SHB, but it is possible that absence of SHB may perturb the signaling response in this context, thus retarding oocyte growth. The present data do not directly address the issue whether ovulation is affected by *Shb* gene inactivation or not. Meiosis I will be completed around this stage, and the loss of appropriate synchronization of meiosis I reentry and subsequent polar body extrusion could suggest some abnormality at this stage as well, although explanations not directly involving ovulation may also account for the observed aberrations.

We also confirm the previously observed transmission ratio distortion of the *Shb*-null allele in oocytes from +/− females. The precise mechanisms remain unresolved, but probably relate to the loss of synchronization of meiosis I completion and the behavior of oocytes in immediate succession of this process.

It should also be noted that some of the effects of *Shb* deficiency on oocyte development are observed in mice carrying the heterozygous mutation. The vascular phenotype was similarly present in the absence of one *Shb* allele and it is thus apparent that haploinsufficiency characterizes certain responses to *Shb* mutations.

In isolated oocytes, abnormal signaling was observed, indicating that the absence of SHB will have direct effects on oocyte function. Enhanced ERK and RSK activity was observed in isolated oocytes and this was paralleled with increased rpS6 phosphorylation. RSK is one of the many kinases that can phosphorylate rpS6 and since RSK is downstream of ERK, a plausible signaling pathway that is operating in the absence of SHB is ERK/RSK/rpS6. In a recent paper, PTEN ablation caused premature activation of the primordial follicle pool, due to enhanced Akt signaling and rpS6 phosphorylation [7]. We did not observe in the present study any effect of *Shb* deficiency on Akt-activation, and thus the alternative ERK/RSK signaling scheme seems more likely to account for the increased rpS6 phosphorylation. Absence of *Shb* shows a phenotype that is clearly distinct from PTEN deficiency, but two similarities are apparent. Both cause premature ovarian development and in both increased rpS6 phosphorylation is apparent. However, the changes in the *Shb* knockout are more moderate, and this could relate to the activation of the alternate (ERK/RSK) signaling pathway causing a moderate increase in rpS6 phosphorylation, contrary to the PTEN-deficiency in which dramatic effects were observed.

One possibility is that absence of SHB will influence basal signaling activity without affecting the response to ligand activation. This is in agreement with a previous study on endothelial cells isolated from *Shb* knockout mice in which increased basal ERK, focal adhesion kinase and p38 MAPK activity was noted [17]. Completion of meiosis requires ERK activity [26], but the relationship between ERK-signaling and meiosis I is less apparent. However, several recent publications suggest that ERK-activation is involved in the resumption of meiosis I [27], [28], [29]. It is therefore likely that increased basal ERK-activity is related to the unsynchronized pattern of polar body extrusion in SHB-depleted oocytes. In addition, Src-family kinases have been shown to exert an influence on meiosis I and II in mouse oocytes [30]. SHB has previously been found to interact with Src-family kinases [8], and loss of such an interaction might contribute to the presently observed abnormalities.

Abnormal blastocyst morphology in *Shb* −/− embryos was observed and this could hamper efficient implantation and further embryo development. Considering that no *Shb* −/− mice were born on the C57Bl/6 background, it seems likely that the morphological aberrations of *Shb* knockout blastocysts are more severe in this strain of mice. The defects in blastocyst morphology presently observed appear to be related to the genotype of the embryo rather than to the genotype of the egg. Thus, despite the abnormalities in oocyte maturation, the oocytes that ovulate and become fertilized seem to harbor no intrinsic deficiency.

The impairments presently observed are quantitative rather than absolute since *Shb* −/− mice are able to reproduce on a mixed genetic background (FVB/J-C57Bl/6-129Sv). This raises the possibility that the signaling defects presently noted might have clinical relevance and it might be worthwhile genetically screening patients with comparable reproductive disorders. We can also infer that interfering with SHB signaling is a potential strategy for manipulating oocyte maturation with a view to enhancing or controlling fertility.

## Materials and Methods

### Animals

The *Shb* knockout mouse was generated, maintained on a mixed genetic background (FVB/N, C57BL/6 and 129/SvJ) and genotyped as described [16]. *Shb* +/− offspring was obtained by mating wild-type mothers with knockout fathers and the converse applied to the *Shb* −/+ offspring. The animals were housed under controlled lighting (photoperiod 06:00 to 18:00 h) and provided with commercial food pellets and tap water *ad libitum*. The experiments were approved by the animal ethics committee of Uppsala University (approval number C44/9).

### Follicle numbers in adult ovaries

Ovaries from *Shb*+/+, *Shb*+/− and *Shb*−/− female mice were collected at random stages of the estrous cycle after euthanasia at ages 1, 6 and 12 weeks (n = 5–10 per group). They were fixed for 24 h in Bouin's fixative, embedded in paraffin wax, sectioned at 5 µm and stained with hematoxylin and eosin. The follicles were counted in every tenth section using the oocyte nucleus as a marker and classified according to the shape and number of layers of somatic cells surrounding the oocyte [31]. Primordial follicles had a small oocyte with flattened pregranulosa cells, primary follicles had a growing oocyte with one layer of cuboidal cells, secondary follicles had multiple layers of granulosa cells and antral follicles contained an antral cavity. For counting primordial follicles Abercrombie's formula was used to correct for overcounting [32]. Ovarian volumes were determined on embedded and serially sectioned ovaries. For each ovary, the largest section corresponding to the estimated geometrical center of the ovary was chosen to measure the major and minor axis of that structure. The z-value was calculated in accordance with the number of sections (each 5 µm) from the beginning of the ovary to that section. The volume was then calculated assuming an ellipsoid structure according to the formula 4π/3*(minor axis/2)*(major axis/2)*(z).

### Fetal oocyte numbers

Fetuses 18.5 days post conception (dpc) were generated in which the day of mating (vaginal plug) was recorded as 0.5 dpc. On 18.5 dpc the gravid mice were killed and ovaries were dissected from female fetuses for counting the number of oocytes or for ovarian culture. Ovaries from five fetuses for four different *Shb* genotypes (*Shb*+/+, *Shb*+/−, *Shb*−/+ and *Shb*−/−) were collected and the oocytes were counted in 5 µm sections (prepared as above), scoring diplotene and pachytene stages separately [33].

*Ovarian culture-* Ovaries from 18.5 dpc fetuses were cut into quadarants of approximately equal size for culture on Millicell-CM Biopore membranes (pore size 0.4 µm, Millipore, Billerica, MA) in DMEM:HAM-F12 (1∶1) (Gibco, Grand Island, NY) supplemented with 15 mM HEPES and 80 µg/ml gentamicin (Gibco, Grand Island, NY) as previously described [34], [35]. The membrane inserts floated on 0.3 ml of culture medium in 24-well tissue culture plates (Nunc, Roskilde, Denmark) cultured at 37°C, 5% CO_2_. The medium was changed every 24 h and cultures continued for 7 days. Afterwards the ovaries were fixed in Bouin's fixative for 1 h, dehydrated and paraffin-embedded for sectioning and staining.

### Mature oocyte analyses and *in vitro* fertilization (IVF)

Mice 6–12 weeks old were stimulated with 5 IU of pregnant mares' gonadotropin (PMSG, Folligon; Intervet, The Netherlands) intraperitoneally (i.p.). Within 46–48 h after PMSG injection, mice were injected with an ovulatory dose of 5 IU of human chorionic gonadotropin, i.p. (hCG; Pregnyl; Organon, The Netherlands). Mice were sacrificed 14–24 h later for collecting oocytes from the oviducts in M2 Media (Sigma-Aldrich, Sweden AB). The oocytes were scored for polar body extrusion at 14 and 24 h after hCG.

Another group of mice the same age was injected i.p. only with 5 IU of PMSG and killed 42 h later for harvesting oocytes from the ovaries using the point of a scalpel to incise the tissue to make pieces <1 mm^3^ [36]. This procedure generates oocytes devoid of cumulus cells, which is a prerequisite for accurately scoring polar body extrusion and oocyte progression to the germinal vesicle breakdown (GVBD) stage. No apparent morphological differences besides the presence of first polar body were noticed between the different genotypes at this stage. The oocytes were washed in M2 medium and those that looked healthy, shiny and spherical were transferred to a drop of M16 medium (Sigma-Aldrich, Sweden AB) covered with mineral oil in which they were cultured for 2, 8, 16, 26 or 40 h. GVBD and first polar body extrusion were scored [36]. To prepare metaphase I stages, oocytes were cultured for 5.0–5.5 h and then treated with 0.3% sodium citrate for 30 min and fixed with freshly prepared methanol: acetic acid (3∶1). Fixed oocytes were stained with Giemsa solution for 1 min, washed and dried in air for visualizing chromosomes.

For IVF were female mice aged 4–6 weeks of three genotypes (n = 2 per group for each experiment, ie *Shb*+/+, *Shb*+/−, *Shb*−/−) injected with 5 IU of PMSG followed by hCG, as described above. They were sacrificed 14 h after the second injection and oocytes with associated cumulus cells were harvested from the oviducts in DMEM Medium. Shortly before sperm was isolated from the epididymides and the vas deferentia from a *Shb*+/+ male mouse and incubated for 10 min before aliquoted to fertilization dishes (see http://cryo.jax.org/ivf.html for details of method). IVF was done in DMEM medium and after 4 h incubation, the eggs were washed to remove excess sperm and transferred to microdrops of 50 µl embryo culture medium (KSOM) containing 20 fertilized oocytes for an additional 24 h culture period under oil allowing differentiation to the morula stage.

### Blastocyst analysis

Blastocysts from four different breeding groups (*Shb*+/+, *Shb*+/−, *Shb*−/+, *Shb*−/−) were collected on 3.5 dpc from the uteri of females 6 to 12 weeks old mated with a male of the appropriate genotype. The blastocysts were examined briefly before fixing overnight in 2.5% glutaraldehyde in 0.1 M sodium cacodylate buffer (pH = 7.2). They were rinsed in the same buffer, stained with 1% osmium tetroxide, dehydrated in graded alcohols and embedded separately in plastic for sectioning at 3 µm and stained with toluidine blue.

### Western Blot analysis

Ovaries were digested at 37°C in a collagenase A solution (2%, w/v) for 30–60 min (according to rates of follicle release) yielding 500–600 oocytes which were collected in SDS sample buffer and heated for 5 min at 100°C [7]. The proteins were separated by SDS-PAGE and then transferred to Hybond membranes which were blocked in TBS+0.1% Tween 20 supplemented with 5% BSA for 4 h. The membranes were incubated overnight at 4°C with one of nine primary antibodies at 1∶1000. The antibodies were phospho-S6 ribosomal protein (Ser235/236) (Cell Signaling Technology, USA), ribosomal protein S6 (C-8) (Santa Cruz Biotechnology, USA), phospho-Akt (ser473), total Akt, phospho-p90RSK (Thr573), total RSK1/RSK2/RSK3, phospho-ERK, total ERK and β-actin. After washing in TBS+0.1% Tween 20 the membranes were incubated for 1 h at room temperature with 1∶1000 HRP-conjugated goat anti-rabbit IgG or HRP-conjugated goat anti-mouse IgG. They were processed using the chemiluminescence (ECL+) detection system (GE Healthcare, Uppsala, Sweden).

### Statistical analysis

Statistical comparisons were made between groups including both parametric and non-parametric tests as stated in the legends to the figures. Non-parametric tests were used when the populations or differences were not normally distributed.

## Supporting Information

Figure S1 Ratio primary/primordial at 1, 6 and 12 weeks of age and ratio antral/primordial at 6 and 12 weeks. Means ± SEM are given for the same number of observations as in Fig. 1. * p<0.05; ** p<0.01; *** p<0.001 using a Students' t-test.
(2.25 MB TIF)

Table S1 Female mouse weights and ovarian volumes of the different genotypes as indicated at the given ages.
(0.03 MB DOC)
