# Supplementary figures and images for: The Src Homology 2 Domain-Containing Adapter Protein B (SHB) Regulates Mouse Oocyte Maturation
Source: PLoS One. 2010 Jun 16;5(6):e11155. doi: 10.1371/journal.pone.0011155 (PMC2886836; doi:10.1371/journal.pone.0011155)

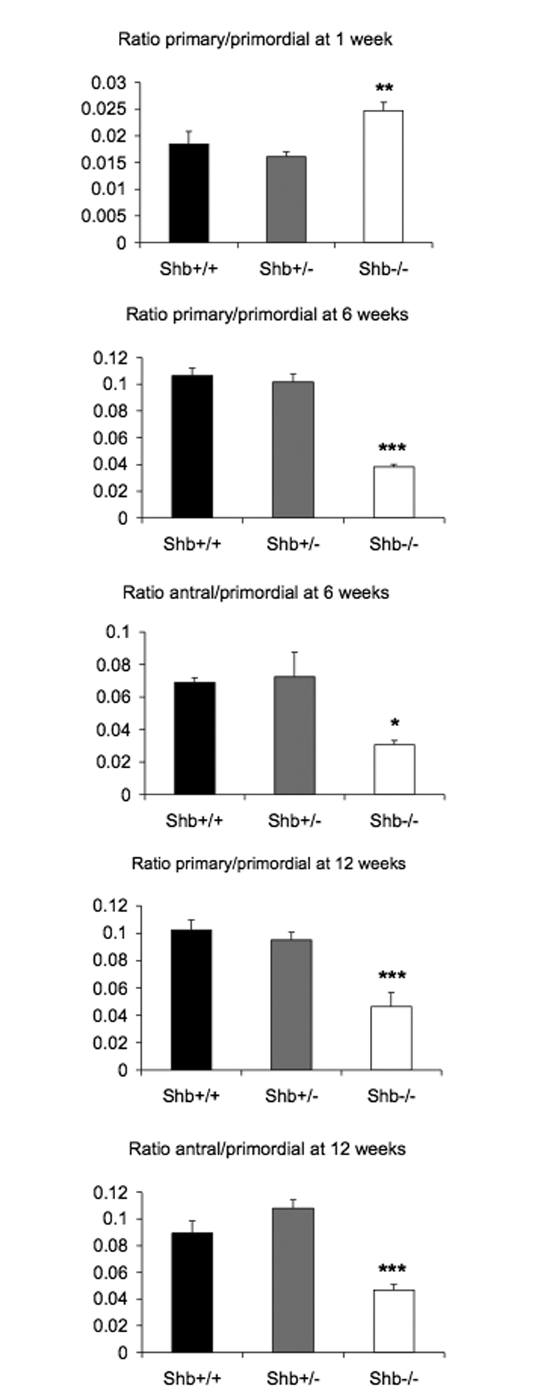

Supplement: Figure S1 — Ratio primary/primordial at 1, 6 and 12 weeks of age and ratio antral/primordial at 6 and 12 weeks. Means ± SEM are given for the same number of observations as in Fig. 1. * p<0.05; ** p<0.01; *** p<0.001 using a Students' t-test. (2.25 MB TIF) [file pone.0011155.s001.tif]
