# Supplementary material for: The Src Homology 2 Domain-Containing Adapter Protein B (SHB) Regulates Mouse Oocyte Maturation
Source: PLoS One. 2010 Jun 16;5(6):e11155. doi: 10.1371/journal.pone.0011155 (PMC2886836; doi:10.1371/journal.pone.0011155)
Supplement: Table S1 — Female mouse weights and ovarian volumes of the different genotypes as indicated at the given ages. (0.03 MB DOC) [file pone.0011155.s002.doc]

| Mouse weight (g) | *Shb*+/+ | *Shb*+/- | *Shb*-/- |
| --- | --- | --- | --- |
| 1 week | 4.67 ±0.30 (n=7) | 3.7 ±0.06 (n=7) | 6.12 ±0.11 * (n=6) |
| 6 weeks | 21.7 ±1.02 (n=5) | 25.0 ±0.63 * (n=5) | 23.6 ±0.31 (n=5) |
| 12 weeks | 28.1 ±0.93 (n=5) | 31.4 ±1.26 (n=6) | 29.7 ±1.88 (n=5) |

| Ovary volume (mm^3^) | *Shb*+/+ | *Shb*+/- | *Shb*-/- |
| --- | --- | --- | --- |
| 1 week | 0.046 ±0.005 (n=7) | 0.054 ±0.003  (n=9) | 0.074 ±0.004 * (n=10) |
| 6 weeks | 2.45 ±0.11  (n=10) | 2.72 ±0.17  (n=10) | 1.73 ±0.10 * (n=10) |
